# Effects of Nanostructures and Mouse Embryonic Stem Cells on *In Vitro* Morphogenesis of Rat Testicular Cords

**DOI:** 10.1371/journal.pone.0060054

**Published:** 2013-03-28

**Authors:** Fei Pan, Lifeng Chi, Stefan Schlatt

**Affiliations:** 1 Institute of Physics, University Münster, Münster, Germany; 2 Centre for Reproductive Medicine and Andrology, Institute of Reproductive and Regenerative Biology, University Münster, Münster, Germany; National Cancer Institute, United States of America

## Abstract

Morphogenesis of tubular structures is a common event during embryonic development. The signals providing cells with topographical cues to define a cord axis and to form new compartments surrounded by a basement membrane are poorly understood. Male gonadal differentiation is a late event during organogenesis and continues into postnatal life. The cellular changes resemble the mechanisms during embryonic life leading to tubular structures in other organs. Testicular cord formation is dependent on and first recognized by SRY-dependent aggregation of Sertoli cells leading to the appearance of testis-specific cord-like structures. Here we explored whether testicular cells use topographical cues in the form of nanostructures to direct or stimulate cord formation and whether embryonic stem cells (ES) or soluble factors released from those cells have an impact on this process. Using primary cell cultures of immature rats we first revealed that variable nanogratings exerted effects on peritubular cells and on Sertoli cells (at less than <1000 cells/mm^2^) by aligning the cell bodies towards the direction of the nanogratings. After two weeks of culture testicular cells assembled into a network of cord-like structures. We revealed that Sertoli cells actively migrate towards existing clusters. Contractions of peritubular cells lead to the transformation of isolated clusters into cord-like structures. The addition of mouse ES cells or conditioned medium from ES cells accelerated this process. Our studies show that epithelial (Sertoli cell) and mesenchymal (peritubular cells) cells crosstalk and orchestrate the formation of cords in response to physical features of the underlying matrix as well as secretory factors from ES cells. We consider these data on testicular morphogenesis relevant for the better understanding of mechanisms in cord formation also in other organs which may help to create optimized in vitro tools for artificial organogenesis.

## Introduction

Following the colonization of the indifferent gonad by primordial germ cells one of the first morphological signs of testicular differentiation is the formation of testis cords. Prior to cord formation, Sertoli cell aggregation is a crucial step to initiate this process [1]. The subsequent migration of cells from the mesonephros is also essential for the formation of testis cords [2].

Sertoli cell cultures have been extensively studied over the last 20 years with special emphasis on the biochemical and genomic effects of hormones and growth factors on Sertoli cell proliferation, metabolism, and differentiation [3]. Coordinated actions of Sertoli and peritubular cells progressing through a morphogenetic cascade are considered fundamental mechanisms during cord formation [4], [5], [6]. The tendency of Sertoli cells to aggregate and to form cord-like structures in culture has been reported for various matrix coated surfaces [6]. While most studies report on the biochemical and genomic effects, few have suggested the possibility that physical and/or mechanical factors affect morphogenesis of testicular cells in vitro. It is still unknown whether Sertoli cells use topographical cues to direct or stimulate morphogenetic events and whether other testicular cell types interfere with this process.

A common approach for controlling cell adhesion to substrates is the introduction of surface topographies [7], [8], [9], [10]. Cells respond to the topographical cues by changing their proliferation, adhesion, migration and orientation. This response is often described as contact guidance [11].

In order to study early interactions between testicular cells and topographical cues, Sertoli cells and peritubular cells isolated from 7-day-old rats were seeded on nanogratingd or flat poly(dimethylsiloxane) (PDMS) substrates. PDMS was chosen due to its inert surface, biocompatibility and frequent use for cell culture studies [12]. The formation of cord-like structures was recorded by time lapse video. We also tested if the addition of OG2 cells (mouse ES cells carrying a GFP reporter gene in the Oct4 promoter region [13], [14]) or conditioned medium collected from OG2 cells affects cord-like structure formation in a dose dependent manner. Eventually, topographical and biochemical cues were combined to test the synergistic effect on cord-like structure formation. Our data elucidate basic steps and mechanisms of testicular tubulogenesis in vitro.

## Materials And Methods

### Surface Preparation

The silicon master substrate (ridge width = groove width = 200 nm/350 nm/5 um, depth = 300 nm; NILT company (Denmark)) was fabricated via laser inference lithography. The master substrate was used as a mold to fabricate replicas by nano-imprinting lithography with Poly(methyl methacrylate) (PMMA). Then, these PMMA substrates were used for the pattern transfer to PDMS substrates [12]. PDMS (Sylgard 184, Dow Corning, Dortmund, Germany) was polymerized by mixing the pre-polymer with the curing agent in a ratio of 10∶1. The mixture was cured on the master substrates at 65°C for 24 h. The substrates were treated with oxygen plasma (50 W for 15 s) to increase surface wettability and cell adhesion. They were sterilized with 70% ethanol, washed with phosphate buffered saline (PBS) and cut into 1 cm^2^ prior to use in the experiments. The surface topography of the PDMS substrates was verified by Atomic Force Microscopy (Digital Instruments, USA, Figure S1).

### Cell Culture

Testicular cells were isolated from 7-day-old rats (CD1 strain) by means of sequential enzymatic digestions as described previously [5]. All animal experiments were performed in accordance with the German law on animal experimentation and were granted by the state authorities of Northrhein Westfalia (8.87-50.10.46.09.053). In brief, after decapsulation and mincing of the testicular tissue, a first digestion was performed in a mixture of collagenase type 4 (1 mg/ml) and DNAse (1 mg/ml) for 10 min at 37°C in a shaking water bath (80 cycles/min). To eliminate Leydig and interstitial cells, the tubule fragments were separated by sedimentation at unit gravity in Dulbecco's Modified Eagle's Medium (DMEM, PAA Laboratories, Pasching, Austria). A second digestion in collagenase type 4 (1 mg/ml), hyaluronidase type 2 (0.5 mg/ml), and DNase (1 mg/ml) for 20 min was performed in order to generate a single cell suspension after repeated pipetting of the cell fragments. Cells were pelleted by centrifugation at 700 rpm for 5 min, then washed and finally resuspended in fresh DMEM to 10^7^ cells/10 ml DMEM. The fragments were seeded on nanostructured and flat PDMS (10^6^ cells/cm^2^) placed on the bottom of 24-well culture plates. The cells were cultured in DMEM containing antibiotics in the absence of serum at 37°C and 5% CO_2_ for up to two weeks. No change of medium was performed during cell culture. Inhomogenous seeding was used on purpose in one setting to achieve different seeding densities of cells inside the same well. Experiments were repeated three times with four independent samples in every condition.

In order to test the effects of mouse ES cells, freshly isolated testicular cells from 7-day-old rats (10^6^ cells/cm^2^) were seeded onto flat PDMS substrates. Different quantities of OG2 cells were added at the time of seeding to reach a final density between 10 and 10^5^ cells/cm^2^. (OG2 cell lines were provided by M. Meisterernst, Institute for Molecular Tumor Biology, University Münster, Germany). All co-culture experiments with OG2 cells were repeated three times with four independent wells for each OG2-cell density.

For preparation of conditioned medium (CM) from OG2 cells, the cells were plated with an initial density of 10^3^ cells/cm^2^ or 10^5^ cells/cm^2^ and were cultured in DMEM for 3 days. Thereafter the medium was collected. Experiments using conditioned medium were repeated three times with four wells representing the different conditions.

### Histology and Immunohistochemistry

At the time of termination of cell cultures the medium was removed and cells were briefly rinsed with PBS. Then the wells were the filled with 4% paraformaldehyde in PBS. Fixation was performed for 15 min at room temperature. After three 5-min washes in PBS, cells were stained immunohistochemically for α-smooth muscle actin (a specific marker for peritubular cells [4]). An anti–α-smooth muscle actin antibody (A2547, dilution 1∶250; Sigma) was used as primary antibody. A goat anti mouse biotin–conjugated secondary antibody (7264, dilution 1∶100; Sigma) and a Strepatividin-peroxidase conjugate (S5512, dilution: 1∶500; Sigma) with Diaminobenzidine (DAB, SigmaFastTM, D4168, Sigma) as substrate was used for visualization of the epitope by generating an insoluble brown precipitate. After counterstaining with hematoxylin, the PDMS samples were removed from the wells and mounted onto microscope slides.

### Live Imaging and Statistical Analysis

For live cell imaging, videos and micrographs were captured with a Zeiss-Axio-Cam Mrm attached to a Zeiss AxioVert 200 with phase-contrast objective. All images were acquired digitally using Zeiss Axio Vision Software (Zeiss).

### Image analysis and statistical analysis

The ImageJ computerized imaging system (http://rsb.info.nih.gov/ij/) was used for image analysis and data processing. In order to measure the orientation of cells, the outline of each cell was marked, and an ellipse was fitted to the outline. Thereafter, the angle between the major ellipse axis and surface gratings was measured. For the flat controls the angle between the major ellipse axis and the image x-axis was measured. At least 100 cells per condition were analyzed. Determination of the orientation of cord-like structures was performed in a similar fashion as for cells with the only difference that large cord-like structures were divided into several segments to obtain the major axis of the ellipse. At least 15 cord-like structures were analyzed per experimental setting to determine changes in the orientation of cord-like structures for each condition. The size of cord-like structures was acquired by circumscribing the outline of individual cord-like structures and calculating the absolute area by the computerized image analysis system. At least 15 cord-like structures were measured in each condition. Cell density was calculated by counting cell numbers in randomly selected areas with the help of the image analysis system All quantitative data are shown as means plusminus standard error. Significance levels were determined using a two-tailed Student's t test. ***p>0.01.

## Results and Discussion

### Alignment of testicular cells on nanostructures

**Figure 1:**
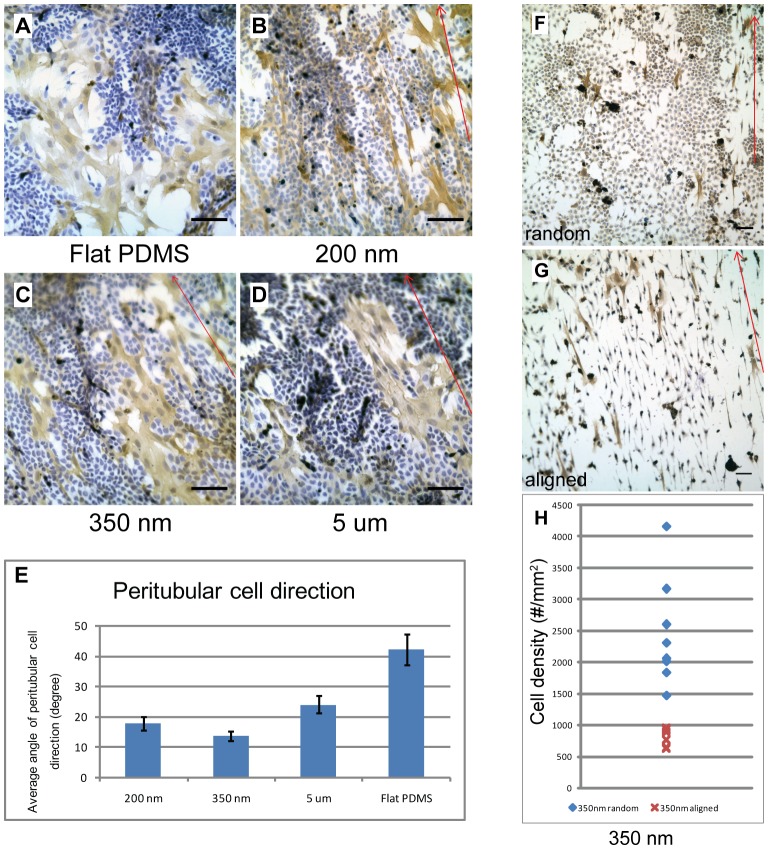
Cell specific and density dependent response of testicular cells to nanogratings. Micrographs showing immunostaining of primary rat (7-day-old) Sertoli cells and peritubular cells. The cells were seeded at an initial density of 10^6^ cells/cm^2^ in DMEM and cultured for one week. Cells were fixed at day 7. Peritubular cells are marked for α-smooth muscle actin (brown precipitate). Nuclei were stained blue with hematoxylin. 1A–D) Cells cultured on flat PDMS substrate (1A), and substrates carrying nanogratings of 200 nm (1B), 350 nm (1C) and 5 um (1D) dimensions. Red arrows indicate the direction of nanogratings. 1E) Quantitatitive analysis of directional changes in peritubular cells on different substrates. All nanogratings evoked changes in the orientation of peritubular cells. No visible influence on Sertoli cells was noted. 1F–H) Cells cultured on 350 nm PDMS substrate after inhomogeneous seeding creating diversity in plating density. Areas of high cell density (1F) revealed a random distribution of Sertoli cells in contrast to an aligned orientation towards nanogratings in low cell density zones (1G) on the same substrate in the same well. Peritubular cells were always oriented in accordance with the direction of the nanogratings. Quantification of cellular orientation in Sertoli cells (1H). Sertoli cell density was determined in randomly selected microscopic frames (as seen in Figs. 1F–G) and the predominant cellular characteristic (either randomly shaped or spindle shaped (aligned)) was recorded for each frame. Seven recordings were performed per experiment and seven independent experiments were analyzed. We established that the threshold to respond to the nanogratings occurred at a density of approximately 1000 cells/mm^2^. Scale bar = 50 um.

The single cell suspensions obtained from immature rats showed the previously described morphogenetic changes of Sertoli and peritubular cells [5], [6]. Prior to the onset of cellular migration after approximately two weeks, the cultures consisted of monolayers of dense Sertoli and sparsely distributed groups of peritubular cells. Neither cell type showed any directional organization on flat PDMS surfaces (Fig. 1A). Exposure of the cells to nanogratings of different dimensions (Figs. 1B–D) revealed a cell-type specific response. Peritubular cells responded to the nanogratings by adapting their cellular axis towards the direction of the nanogratings irrespective of their dimensions. In contrast no obvious response was observed from Sertoli cells which formed extended monolayers with no directional response towards the nanogratings. As peritubular cells responded most intensely to nanogratings with a width of 350 nm (Fig. 1E), we decided to perform all subsequent experiments using this dimension.

To explore the effects of cell density we performed subsequent experiments culturing the cells on 350 nm PDMS substrates after inhomogeneous plating. Figs. 1F–G reveal that the orientation of the peritubular cell body was always in line with the nanogratings irrespective of cell density. Sertoli cells responded to nanogratings only at low cell density changing into a spindle-like shape with strict orientation of their cell bodies in the direction of the nanostructures. This obvious change in phenotype occurred below a cell density of 1000 cells/mm^2^ as was estimated by cell counts (Fig. 1H). Generating different cell densities by inhomogeneous plating on the same substrate led to the appearance of both phenotypes in the same well indicating that direct cell-to-cell contacts but not soluble factors were responsible for the obvious morphological change in response to nanostructures. We conclude from this first series of experiments that peritubular cells and Sertoli cells differ in their responsiveness to nanostructures and that Sertoli cells – in contrast to generally sensitive peritubular cells - respond to the presence of nanostructures only at low cell density.

### Effects of nanostructures on cord-like structure formation

**Figure 2:**
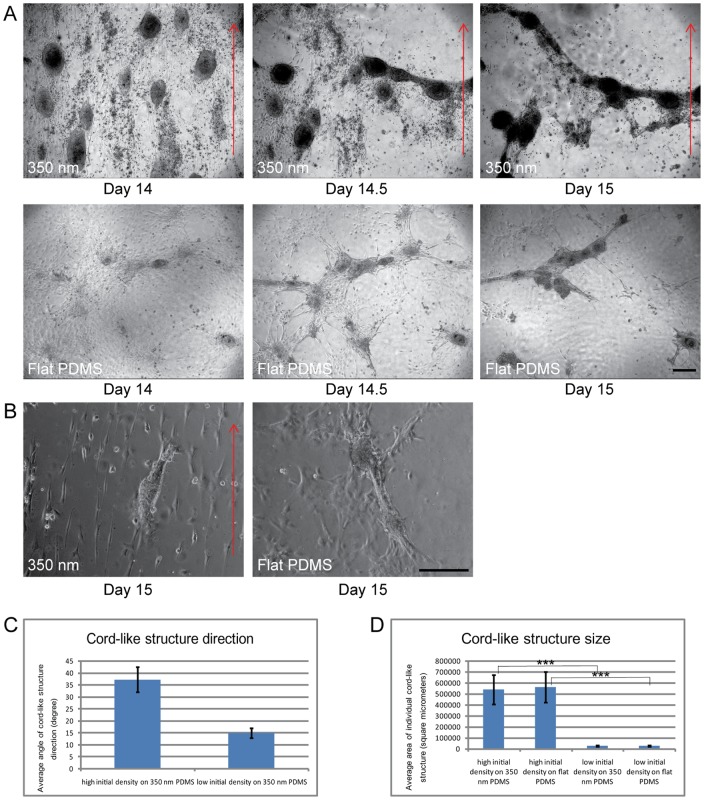
Cord like-structure formation in testicular cell cultures (see also Video S1). 2A) Two series of three micrographs presenting the stages of cord-like structure formation in testicular cells cultured on 350 nm and flat PDMS substrates in DMEM at days 14, 14.5 and 15 of culture. Primary rat (7-day-old) Sertoli and peritubular cells were seeded at an initial density of 10^6^ cells/cm^2^. At two weeks, cultures consisted of confluent mixed monolayers with several Sertoli cell clusters. Imaging was started at day 14 when first indications of cellular migration were observed. During the observation period active movements triggered a change of the confluent monolayers into cord-like structures on both 350 nm (upper panel) and flat PDMS substrates (lower panel). The orientation of cord-like structures did not follow the direction of nanogratings (red arrows). 2B) Testicular cell cultures performed under identical conditions as described for Fig. 2A except that cells were seeded at a lower initial density of 10^5^ cells/cm^2^ either on nanograting (left panel) or flat (right panel) PDMS substrates. Micrographs were taken at day 15 of culture. The orientation of cord-like structures was following the direction of nanogratings (red arrow). Scale bar = 200 um. 2C) Analysis of changes in the direction of cord-like structures seeded at high (10^6^ cells/cm^2^) or low (10^5^ cells/cm^2^) initial density on 350 nm PDMS substrates at day 15. 2D) Analysis of the size of individual cord-like structures seeded at high (10^6^ cells/cm^2^) or low (10^5^ cells/cm^2^) initial density on 350 nm and flat PDMS substrates at day 15.

After extended culture of mixed testicular cell suspensions for about two weeks intense migration and aggregation of Sertoli cells was described previously [5]. We recorded time-lapse videos and still images from our cultures which were initiated when first signs of cellular aggregation were observed. Fig. 2A depicts a series of three micrographs taken at day 14, day 14.5 and day 15 when the cells were either cultured on nanogratings (upper panel) or flat PDMS (lower panel) substrates. At this stage Sertoli cells and peritubular cells had formed dense and complete monolayers with some Sertoli cell clusters. During the first few hours directed cellular migration leads to the opening of several cell-free zones which quickly extend. Migration occurs into the direction of the existing Sertoli cell clusters leading to their further expansion. As can be viewed in the time-lapse video (Video S1) contractions occurring at the edges of the cell free zones generate a re-organization and reunion of Sertoli cell clusters into extended elongate structures. The clusters become slowly connected by continuing contractions. Finally a cord-like structure consisting of several dense Sertoli cell zones is seen stretching over the entire field of view. Afterwards the cord-like structures loose connection to the wells and eventually float in the culture medium. There was no effect of nanogratings to determine the orientation of cord-like structures as no difference was detectable between flat substrates and those with nanostructures. It was not possible to predict the direction of cord-like structures or the extent of cord-like structure formation prior to the initiation of cellular migration. We conclude that aggregation into clusters is an initial process prior to cord formation. Subsequent contractions lead to the formation of cord-like structures by combining clusters and stretching them along an axis which is not guided by nanostructures at high cell density.

As we concluded from our first experimental series that the alignment of Sertoli cells on nanogratings is cell-density dependent, we tested if a low seeding density of 10^5^ cells/cm^2^ reflecting the threshold value estimated in the first series (Fig. 1H), has an effect on the formation of cord-like structures. Fig. 2B depicts the formation of small cord-like structures at day 15 on 350 nm and flat PDMS substrates. The cord-like structures resulting after plating at low density responded to nanogratings. Performing a quantitative analysis a better alignment of cord like structures with the nanogratings was detected when the seeding density was low (10^5^ cells/mm^2^) compared to our standard seeding density (10^6^ cells/mm^2^; Fig. 2C). However the size of cord-like structures was reduced when compared to high cell density seeding (Fig. 2D).

### OG2 cells or conditioned medium accelerate cord-like structure formation

**Figure 3:**
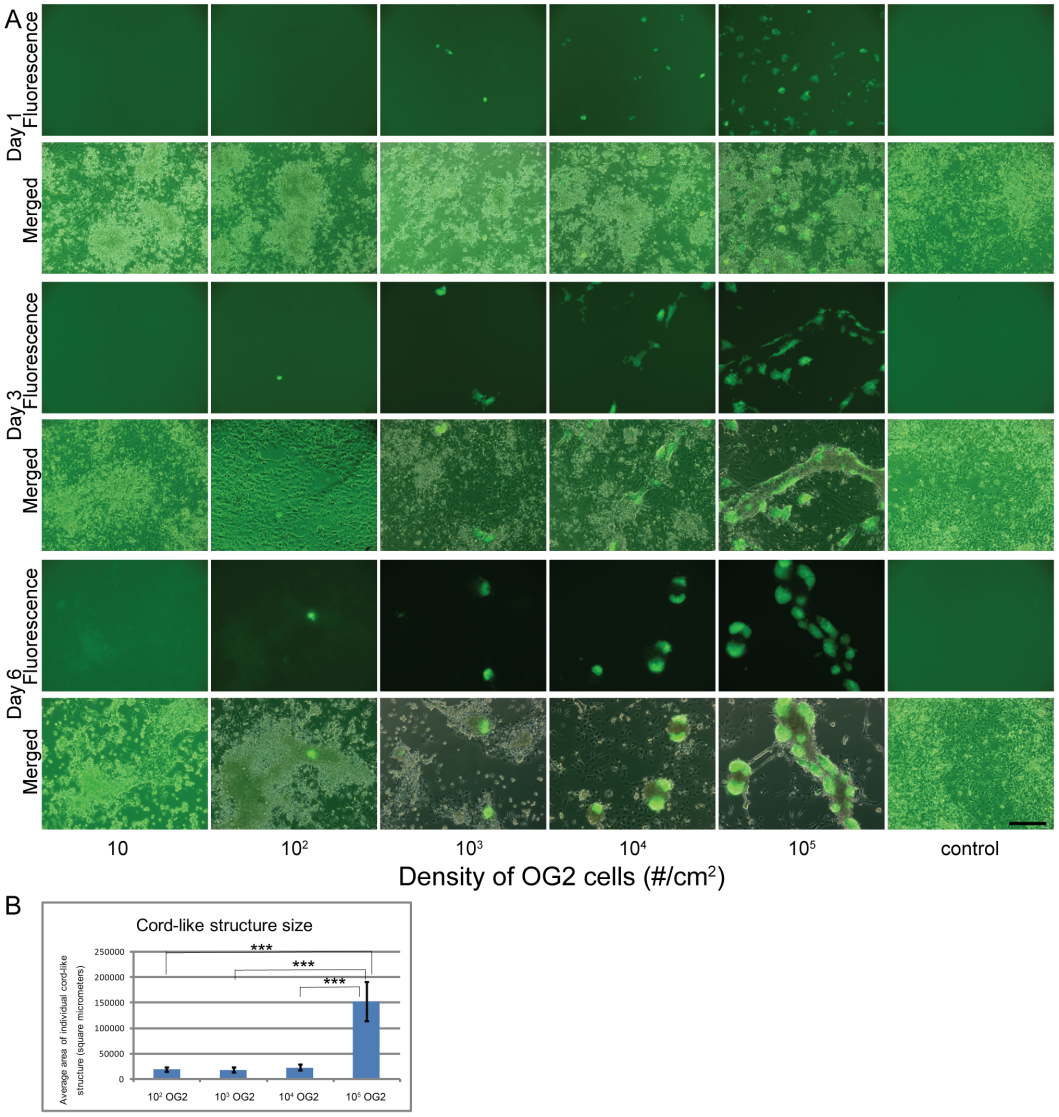
Effects of mouse embryonic stem cells (OG2 cells expressing the GFP-transgene) on cord formation of rat testicular cells. 3A) Micrographs showing immunofluorescence of GFP-signal (upper panels) and merged images (phase contrast plus immunofluorescence) of primary rat (7-day-old) Sertoli cells and peritubular cells seeded at an initial density of 10^6^ cells/cm^2^ on flat PDMS substrates after culture for one day (upper two panels), three days (middle two panels) and six days (lower two panels) in DMEM. A variable number of OG2 cells (10, 10^2^, 10^3^, 10^4^, 10^5^ cells/cm^2^) was added to the primary cells at the initiation of cell cultures. No OG2 cells were added to controls. No obvious change of cellular arrangements was observed at day 1. On days 3 and 6 of culture cord-like structure formation is observed which intensified with increasing numbers of OG2 cells. OG2 cells formed expanding colonies in contact with the cord-like structures. 3B) Analysis of the size of cord-like structures at day 6 of culture. Cord-like structures were significantly larger compared to all other experimental groups depending on the initial density of OG2 cells on flat PDMS substrates at day 6. In the control group and after addition of only 10 OG2 cells no cord like structures were encountered.

The role of mouse embryonic stem cells or factors released from these cells on testis cord formation was tested in another series of experiments. OG2 cells were added during seeding to testicular cells growing on flat PDMS for six days. Fig. 3A reveals the dose-dependent changes at day 1, day 3 and day 6 after different numbers of OG2 cells were added to the culture dish. No obvious changes were recorded at day 1. After three and six days OG2 cells induced a cell-number dependent stimulation of cord-like structure formation. OG2 cells accelerated and intensified the establishment of cord-like structures (Fig. 3A). Determining the size of cord-like structures in different conditions at day 6 revealed that at high concentrations of OG2 cells cord formation was not only accelerated but also resulted in an enlargement of cord-like structures (Fig. 3B). No cord-like structures were observed in control conditions and after addition of only 10 OG2 cells/cm^2^ at day 6. OG2 cells colonized the newly formed cord-like structures and established expanding colonies in contact with the cords.

**Figure 4:**
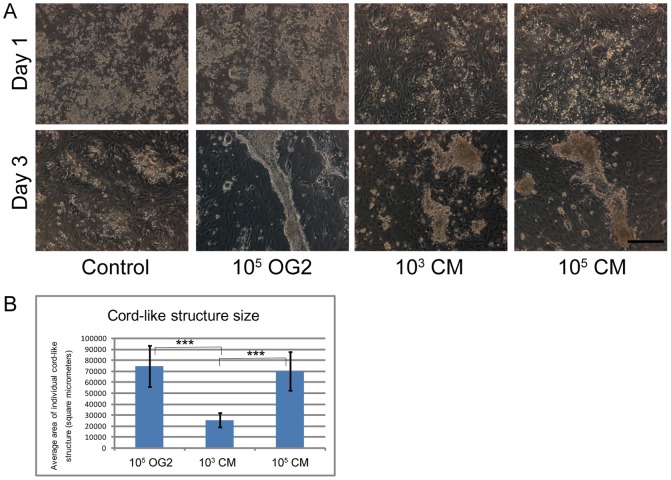
Acceleration of cord-like structure formation by factors released from OG2 cells. 4A) Phase contrast micrographs of primary rat (7-day-old) testicular cells seeded at an initial density of 10^6^ cells/cm^2^ on flat PDMS substrates. Cells were fixed after culture in DMEM for one (upper panel) or three (lower panel) days. Four conditions were created by adding at initiation of cultures either no cells or conditioned medium (control) or 10^5^ OG2 cells/cm^2^, or conditioned medium from 3 day cultures of OG2 with a low density of 10^3^ cells/cm^2^ or high density of 10^5^ cells/cm^2^. At day 1, no obvious changes or differences was visible in cultured cells. At day 3 cellular aggregation and cord-like structure formation was observed after addition of either OG2 cells or both concentrations of conditioned medium. Scale bar = 200 um. 2B) Analysis of the size of cord-like structures at day 3 of culture. Cord-like structures were significantly larger after addition of OG2 cells and conditioned medium from high density OG2 cultures compared to cultures receiving conditioned medium from low density OG2 cultures. In the control group no cord like structures were encountered.

In order to explore if soluble factors released from OG2 cells are sufficient or if direct cell-cell contacts are required for acceleration of cord-like structure formation, we added either OG2 cells or conditioned medium from OG2 cells to the testicular cells (Fig. 4A). In comparison to control conditions, the addition of OG2 cells or conditioned medium enhanced cord-like structure formation in the cultures analyzed at day 3. These data show that soluble factors are responsible for the acceleration of cord formation. Determination of cord size revealed a dose dependent effect of the soluble factors at day 3 of culture (Fig. 4B). The addition of OG2 cells and conditioned medium from a high density OG2 culture generated a similarly strong response while after addition of conditioned medium from low density cultures smaller cord-like structures were encountered.

### Combined action of conditioned medium and nanostructures on cord-like structure formation

**Figure 5:**
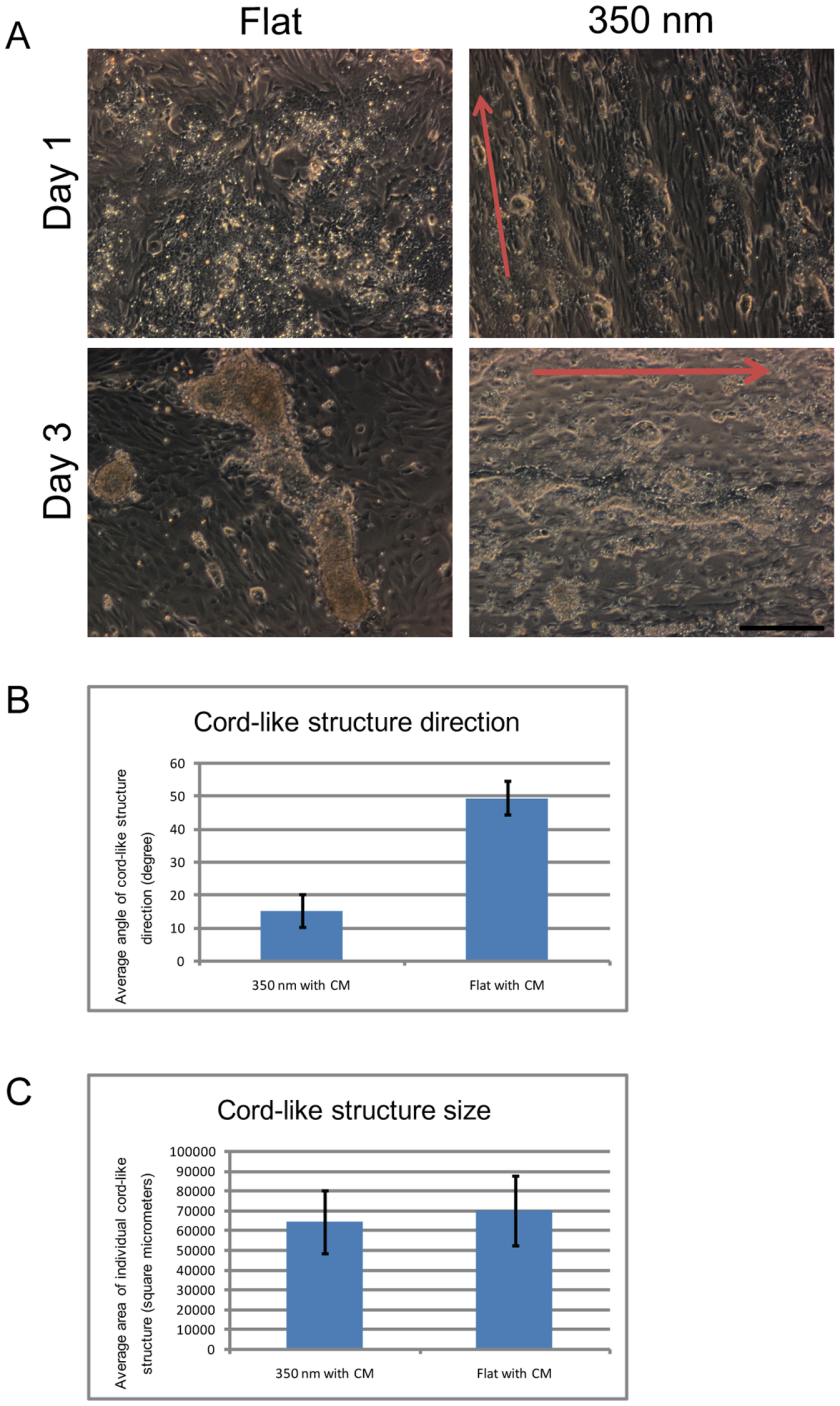
Combined effects of nanostructures and OG2 cell conditioned medium on cord formation of testicular cells. 5A) Phase contrast micrographs of primary rat (7-day-old) testicular cells seeded at an initial density of 10^6^ cells/cm^2^ on 350 nm (right panel) and flat (left panel) PDMS substrates in conditioned medium (Three day culture of OG2 cells at a density of 10^5^ cells/cm^2^) at day 1 (upper panel) and day 3 (lower panel) of culture. At day 1 orientation of cells in the direction of nanogrids was visible when compared to flat PDMS substrates. At day 3 of culture cellular aggregation and cord-like structure formation occurred on both flat and 350 nm PDMS substrates. The direction of cord-like structure was aligned with nanogratings on 350 nm PDMS substrates but not on flat PDMS substrates. Red arrows indicate the direction of nanogratings. Scale bar = 200 um. 5B) Analysis of the direction of cord-like structures in conditioned medium and after exposure to 350 nm or flat PDMS substrates at day 3 of culture. The quantitative results confirm the microscopic observation that nanogratings have an impact on the orientation of cord-like structures. Cord-like structures on flat PDMS show a random orientation. 5C) Analysis of the size of cord-like structures at day 3 of culture. Cord-like structures were of similar size irrespective of the exposure to nanogratings.

Finally we intended to explore if the addition of OG2-conditioned medium in combination with nanogratings can be used to accelerate formation and direction of in vitro generated cord-like structures. After exposure of testicular cells to OG2 cell conditioned medium and culture on 350 nm PDMS substrates, cord-like structures became oriented towards the direction of the nanogratings at day 3 of culture (Fig. 5A, B). Cord like structures were of similar size in both conditions (Fig. 5C) but did not show any preferred directional preference in controls (Fig. 5A). Using these conditions we were able to control and direct the formation of testicular cord-like structures.

## Conclusions

Nanostructures exert strong cues on peritubular cells and Sertoli cells. However, their effects on Sertoli cells vanishes at high cell density indicating that crosstalk among densely packed Sertoli cells overcomes the effects of nanostructures in the underlying matrix. The presence of ES cells or conditioned medium accelerates cord-like structure formation in a dose dependent manner indicating that these cells provide soluble factors promoting testicular organogenesis. Our experiments reveal that an orchestration of external stimuli from the underlying matrix and soluble factors released from surrounding cells crosstalk to control the formation of cord-like structures. The concerted interaction of ES cells, epithelial cells (Sertoli cells) and mesenchymal cells (peritubular cells) is crucial to develop cords under in vitro conditions. Our observations in cells from the postnatal testis may have implications for the mechanisms leading to cord formation in many other organs during early embryogenesis.

## Supporting Information

Figure S1 **Atomic force microscopy (AFM) micrographs of nanostructured PDMS.** Regular gratings of defined dimension are visible (yellow color representing the ridge and brown color representing the groove of the structures). Width of each different nanograting is indicated. A schematic drawing of a cell growing on a nanostructured surface of approximately 5 um reveals cellular exposure to the underlying PDMS matrix.
(TIF)

Video S1 **Time lapse video sequence revealing the process of cord formation in rat testicular cells.** Primary rat (7-day-old) Sertoli cells and peritubular cells were grown at an initial density of 10^6^ cells/cm^2^ on nanostructured PDMS (nanogratings of 350 nm width) in DMEM. At two weeks cultures consisted of confluent monolayers with some Sertoli cell clusters. Time lapse imaging was started when first cellular movements were observed at day 14. The observation was continued for 24 hours. We recorded 720 image frames in total and converted these into a movie sequence of 144 seconds with 5 frames per second. One second in the time lapse video is equal to 10 minutes real time.
(MP4)
