# Supplementary figures and images for: Effects of Nanostructures and Mouse Embryonic Stem Cells on In Vitro Morphogenesis of Rat Testicular Cords
Source: PLoS One. 2013 Mar 28;8(3):e60054. doi: 10.1371/journal.pone.0060054 (PMC3610873; doi:10.1371/journal.pone.0060054)

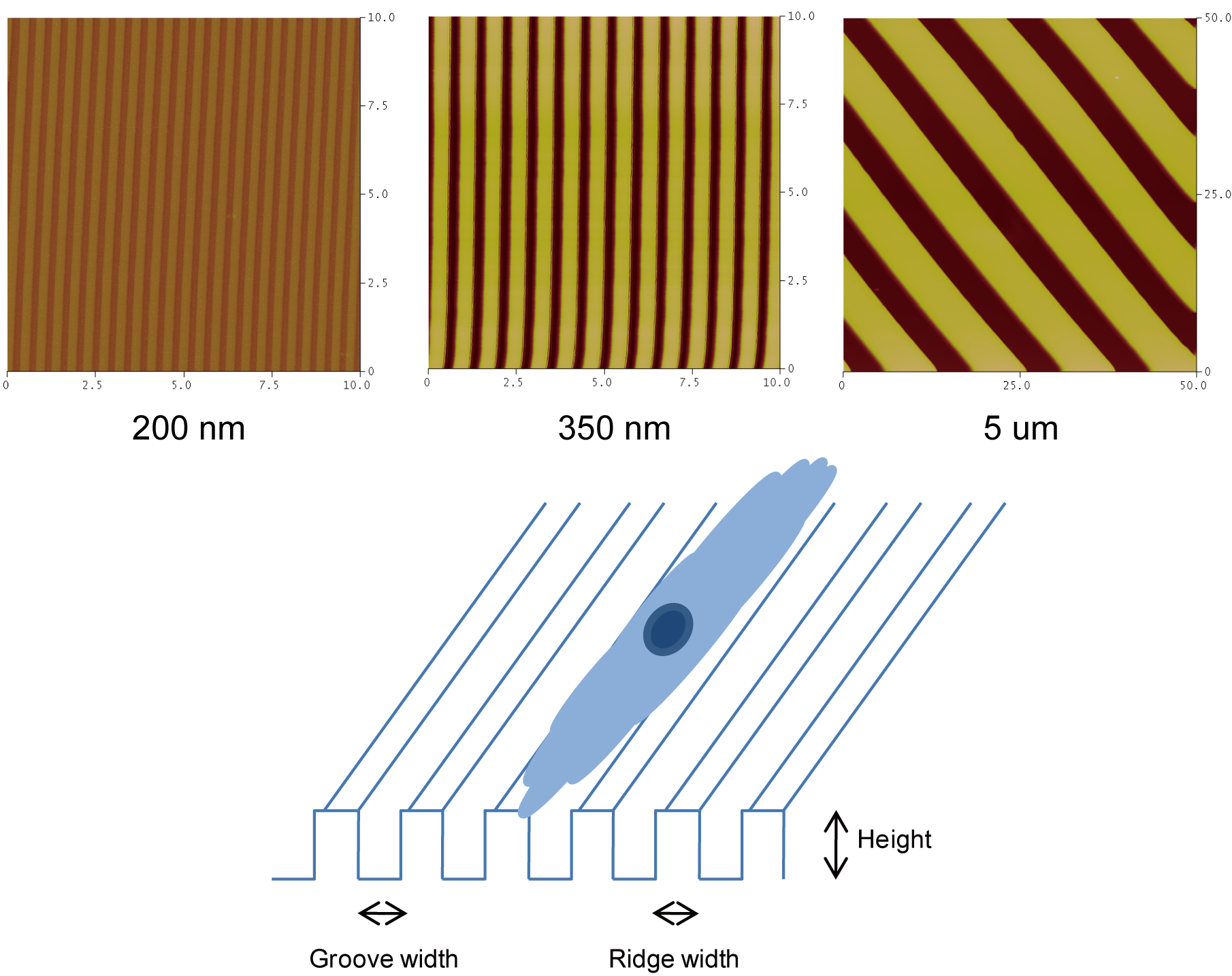

Supplement: Figure S1 — Atomic force microscopy (AFM) micrographs of nanostructured PDMS. Regular gratings of defined dimension are visible (yellow color representing the ridge and brown color representing the groove of the structures). Width of each different nanograting is indicated. A schematic drawing of a cell growing on a nanostructured surface of approximately 5 um reveals cellular exposure to the underlying PDMS matrix. (TIF) [file pone.0060054.s001.tif]
